# Women in Otolaryngology

**DOI:** 10.1186/1916-0216-43-14

**Published:** 2014-06-12

**Authors:** Jonas T Johnson

**Affiliations:** 1 Department of Otolaryngology, University of Pittsburgh School of Medicine, Eye and Ear Institute, Suite 500 203 Lothrop Street, Pittsburgh, PA 15213, USA

**Keywords:** Gender disparity, Academic medicine, Women in otolaryngology, Glass ceiling

## 

Information collected and disseminated through the American Association of Medical Colleges (AAMC) documents that women are currently entering medicine at a rate very similar to men. As recently as 1985, almost 75% of medical student graduates were male. Not surprisingly, men occupy appointments as full professors and associate professors far more often than women. On the surface of it, this may be reflective of the relatively smaller pool of women in medicine just two decades ago. However, the slope of growth in rank of women in surgery as recently as 2009 does not (11.89) equal the continuing growth in rank of men in surgery (42.79) [1].

A number of studies have been accomplished which suggest that gender may limit chances for promotion. Sexton et al. [2] surveyed surgical faculty at their institution. Women were far more likely to strongly agree or agree with the statement, “my gender limits my chance for promotion”. These data seem to sustain the observation that there does, in fact, exist a “glass ceiling” in academic surgery [3]. Medscape (2013) [4] supports the situation that was reported in April 2013 that male physicians, on average, are paid $60,000 per year more than women.

The legal landscape in the United States holds that all individuals have equal protection under the law. Accordingly, it seems peculiar that we observe increasing numbers of female medical students and residents with no real change in representation of academic women surgeons at each faculty rank in the last 15 years. Additionally, the proportional representation of female full professors is unchanged over 35 years. Failure to fairly promote women into senior academic positions represents a lost opportunity to benefit from talent of all academic physicians.

A prospective randomized trial was undertaken by Moss-Racusin et al. [5] in which identical credentials were randomly submitted for evaluation to a science faculty of 127 individuals. The credentials were identical except that one was representative of a female applicant, while the other was male. The chosen names were pretested as equivalent in “likeability”. The rated competence, “hire ability”, and mentoring by gender condition favored the male applicant over the identically credentialed female applicant, and the evaluators consistently suggested that a higher salary be conferred on the male applicant.

Some studies have compared academic contributions of women to those of men. Eloy et al. [6] have studied gender disparities and scholarly productivity in otolaryngology. Using the H-index for comparisons, they do note that women have less output earlier in their career, however, at senior levels, women actually exceed men. Duch et al. [7] point out that “when the time is long, pursuing an academic position is highly risky”. Bergeron et al. [8] report that analysis of publications in otolaryngology since 1978 show significant and steady increases in female authorships.

It is appropriate to ask, do women lack adequate mentors in the surgical fields? There seems to be some distinctive needs of women in terms of the constraints of traditional role, and there may be a scarcity of senior role models in academia. Traditional gender roles may be incompatible with the demands of family commitments and may constrain career-related choices. Additionally, there are still reported manifestations of sexism in the surgical workplace which includes inappropriate sexual behavior, differential access to career-promoting experiences, discrimination in tenure and promotion decisions, and lack of serious attention to female candidates in the search process.

The gender gap in surgical subspecialty training was explored by Grandis et al. [9]. A questionnaire was sent to all female members of the American Academy of Otolaryngology-Head and Neck Surgery (n = 502). A similar survey was sent to male members matched for geographic region, practice type and years since completing training in a 2:1 match. The demographics demonstrate that similar portions of men and women were married and had advanced degrees. Men and women otolaryngologists reported similar numbers of children. Women were more likely to be divorced, more likely to be married to a physician, and more likely to report (14% v 3%) that their spouse’s career was most important in their family. Men were more likely to be fellowship-trained (38.5% v 31%). Men consistently reported higher income (averaging $40,000 per year more than women), but men also reported working >60 hours per week and more time spent in the operating room.

By way of contrast, women were far more likely to run the home and spend greater than 20 hours per week on home care work. Women are more likely to be involved in evening and weekend childcare, and women are far, far more likely to be primarily responsible for the care of a sick child (89% v 14%). When asked about career satisfaction, women report more often that career interferes with personal life, while both men and women are equally happy with family, marriage, and health.

Women are more likely to state that female medical students need female role models and, interestingly, all respondents were less likely to encourage women to pursue surgery as a career choice. Women are also more likely to report having experienced sexual harassment sometime during their career (32% v 3%), and women feel that their sex hinders their career (17% v 5%).

Is it possible that there is truly a “glass ceiling” in academic surgery? These considerations may be deeply embedded in unconscious gender bias assumptions. Organizational cultures favor men through mentoring and networking, and there is disparity in family responsibility and the relative lack of role models for women.

Jordan Cohen, past president of the AAMC, suggested, “Cultivating diversity in our faculty and in our leadership is an indispensable strategic instrument for meeting the challenges that academic medicine faces in the 21^st^ century. Grooming women for leadership positions and eradicating the barriers currently impeding their success are essential components of this strategy. Those institutions that fail to seize the advantages offered by elevating talented women to positions of power are destined to be eclipsed by those that do”.

The leadership of academic surgical organizations of the present has the opportunity to change the culture. Mistakes of the past can be relegated to the past as we work prospectively into the future to provide more flexible work and training schedules, consider job sharing and part-time opportunities, and implement formal mentoring programs throughout training.

Malcolm Gladwell speculates, in *Blink,* that we all tend to make important judgments based upon an instant of exposure. We all should take precaution to avoid this having a discriminatory impact in the workplace.

## Competing interests

The author has no competing interests.

## Authors’ information

Jonas T. Johnson, M.D., is the Dr. Eugene N. Myers Professor and Chair of Otolaryngology, Department of Otolaryngology, University of Pittsburgh School of Medicine, Pittsburgh, PA. This manuscript is the result of his keynote speech at the Canadian Society of Otolaryngology-Head and Neck Surgery 67^th^ Annual Meeting, Banff, Canada, June 2–4, 2013.
